# Mechanisms of Multidrug Resistance in Cancer Chemotherapy

**DOI:** 10.3390/ijms21093233

**Published:** 2020-05-02

**Authors:** Karol Bukowski, Mateusz Kciuk, Renata Kontek

**Affiliations:** Department of Molecular Biotechnology and Genetics, Faculty of Biology and Environmental Protection, University of Lodz, 12/16 Banacha St., 90-237 Lodz, Poland; mateusz.kciuk@unilodz.eu (M.K.); renata.kontek@biol.uni.lodz.pl (R.K.)

**Keywords:** cancer, multidrug resistance, chemotherapeutics, inhibitors, P-glycoprotein, drug metabolism, growth factors, DNA repair, epigenetic alterations, microRNA

## Abstract

Cancer is one of the main causes of death worldwide. Despite the significant development of methods of cancer healing during the past decades, chemotherapy still remains the main method for cancer treatment. Depending on the mechanism of action, commonly used chemotherapeutic agents can be divided into several classes (antimetabolites, alkylating agents, mitotic spindle inhibitors, topoisomerase inhibitors, and others). Multidrug resistance (MDR) is responsible for over 90% of deaths in cancer patients receiving traditional chemotherapeutics or novel targeted drugs. The mechanisms of MDR include elevated metabolism of xenobiotics, enhanced efflux of drugs, growth factors, increased DNA repair capacity, and genetic factors (gene mutations, amplifications, and epigenetic alterations). Rapidly increasing numbers of biomedical studies are focused on designing chemotherapeutics that are able to evade or reverse MDR. The aim of this review is not only to demonstrate the latest data on the mechanisms of cellular resistance to anticancer agents currently used in clinical treatment but also to present the mechanisms of action of novel potential antitumor drugs which have been designed to overcome these resistance mechanisms. Better understanding of the mechanisms of MDR and targets of novel chemotherapy agents should provide guidance for future research concerning new effective strategies in cancer treatment.

## 1. Introduction

Cancer is responsible for about 1 in 6 deaths worldwide. It is the second leading cause of death globally, with 8.7 million deaths in 2015 [1]. Factors that are associated with elevated risk of cancer are tobacco use (22% of cancer deaths), lack of physical activity, alcohol use, low vegetable and fruit intake, and high body mass index. These factors are thought to be responsible for approximately one third of cancer deaths. Breast, cervical, lung, thyroid, and colorectal cancers are the most common types of cancer in women, while prostate, lung, colorectal, liver, and stomach cancer are the most common among men [2]. Despite the fact that there are several different methods of cancer treatments, including radiation therapy, surgery, immunotherapy, endocrine therapy, and gene therapy, chemotherapy still remains the most common method of cancer healing. In this paper, we have presented the latest data on the mechanisms of cellular resistance to chemotherapy and chemotherapeutics currently used in clinical treatment as well as on the mechanisms of action of novel potential anticancer agents which have been designed to overcome these resistance mechanisms.

## 2. Types of Chemotherapeutics

**Figure 1:**
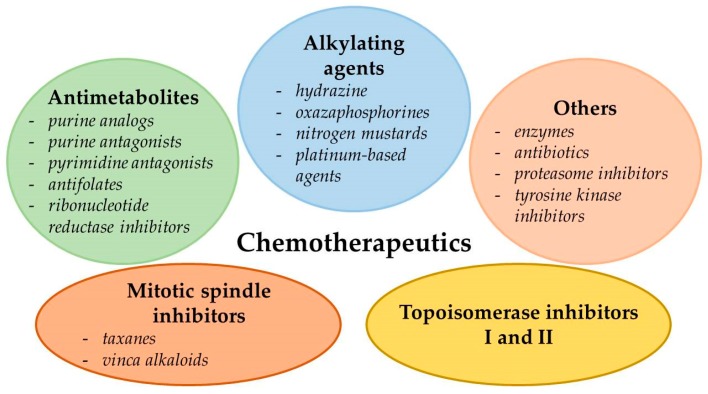
Classification of commonly used chemotherapeutics depending on their mechanism of action [7,8,9].

Chemotherapeutics can be divided into two classes depending on/regarding their origin. They can either be plant-derived (extracted from plants) [3,4] or of synthetic origin [5,6]. Depending on the mechanism of action, they can be divided into alkylating agents, antimetabolites, topoisomerase inhibitors, mitotic spindle inhibitors, and others (Figure 1) [7,8,9].

Alkylating agents include the oxazsaphosphorines (cyclophosphamide and ifosfamide); nitrogen mustards (busulfan, chlorambucil, and melphalan); hydrazine (temozolomide); platinum-based agents (cisplatin, carboplatin, and oxaliplatin) [7]; and novel, still under investigation OFF-ON-type alkylating agents such as vinyl-quinazolinone (VQ) [10]. Chemotherapeutics belonging to this class of molecules create either inter or intra-strand cross links or transfer alkyl groups to the guanine residues of DNA, which results in mispair formation in DNA bases and prevents strand separation during DNA synthesis [7,8].

Antimetabolites can be divided into several groups: pyrimidine antagonists (cytarabine, 5-fluorouracil (5-FU), gemcitabine, and capecitabine), purine antagonists (fludarabine), purine analogs (6-mercaptopurine, azathioprine, and cladribine), antifolates (methotrexate, pemetrexed, and pralatrexate), and ribonucleotide reductase inhibitors (hydroxyurea). These anticancer drugs interfere with essential biosynthetic pathways, disturb the DNA/RNA synthesis, or cause the formation of DNA strand breaks through inhibition of particular enzymes (dihydrofolate reductase, ribonucleotide reductase, and DNA polymerase) or incorporation of false structural analogues of pyrimidine/purine into DNA [5,7,8].

Topoisomerase I inhibitors (irinotecan and topotecan) and topoisomerase II inhibitors (etoposide; teniposide; and anthracyclines, e.g., idarubicin, daunorubicin, and doxorubicin (DOX)) inhibit topoisomerases activities involved in replication of DNA and cause DNA strand breaks [7,8,11,12].

Mitotic spindle inhibitors such as taxanes (docetaxel and paclitaxel) and vinca alkaloids (vincristine (VCR) and vinblastine) modify the function/formation of spindle microtubules by inhibition of nuclear division (mitotic arrest in metaphase), leading to cell death [7,8]. Recently, Peng et al. [6] demonstrated that one of the newly synthesized N-carbonyl acridines inhibited tubulin polymerization, presenting high antiproliferative activity against human mammary gland/breast cancer cells MB-468 (half-maximum inhibitory concentration—IC50—value comparable to colchicine and paclitaxel).

Other chemotherapeutic agents, including some enzymes (l-asparaginase), proteasome inhibitors (bortezomib), tyrosine kinase inhibitors (imatinib and erlotinib), and antibiotics (bleomycin, actinomycin D, and anthracyclines), are characterized by non-homogenous mechanisms of action. While l-asparaginase cleaves the amino acid l-asparagine essential for normal cell metabolism, bortezomib drives the cell to apoptotic death by inhibition of apoptotic protein degradation. Imatinib and erlotinib inhibit tyrosine kinases activities involved in multiple intracellular pathways associated with receptor-mediated growth signaling, leading to cellular dysfunction and subsequent cell death. Bleomycin, an antibiotic, induces formation of free radicals that cause DNA damage and the cell cycle arrest in G2 phase. Another anticancer agent, actinomycin D, intercalates into DNA and interferes in DNA transcription. Anthracyclines exhibit anti-proliferatory effects in the abovementioned processes and inhibit topoisomerase II activity [7].

## 3. The Problem of Drug Resistance in Cancer Chemotherapy

**Figure 2:**
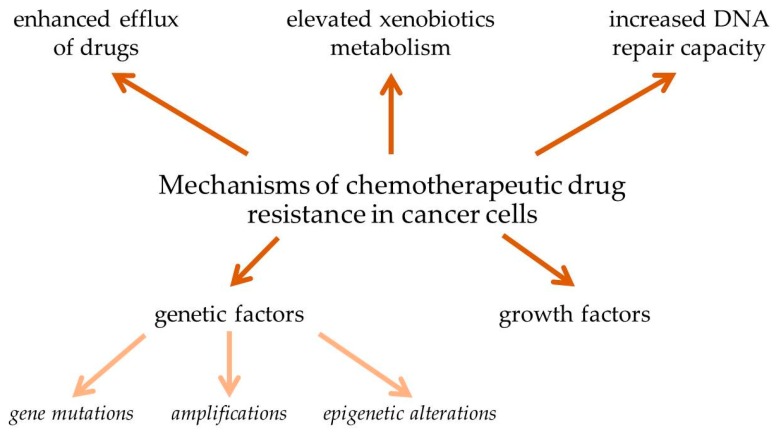
Mechanisms of chemotherapeutic drug resistance in cancer cells [9,13,14,15,16].

Statistical data shows that over 90% mortality of cancer patients is attributed to drug resistance. MDR of cancer cells during chemotherapy can be associated with a variety of mechanisms, including enhanced efflux of drugs, genetic factors (gene mutations, amplifications, and epigenetic alterations), growth factors, increased DNA repair capacity, and elevated metabolism of xenobiotics (Figure 2). Each of these mechanisms leads to reduction of the therapeutic efficacy of administered drugs, causing more difficulty in tumor treatment [9,13,14,15,16].

### 3.1. Enhanced Efflux of Drugs

ATP-binding cassette (ABC) proteins such as P-glycoprotein (P-gp)/ATP-binding cassette subfamily B member 1 (ABCB1) or Breast Cancer Resistance Protein (BCRP) present in the cell membrane are responsible for regulation of distribution, absorption, and excretion of a variety of chemical compounds. Because these proteins protect cells from death caused by high intracellular drug concentration, they can also interfere with drug administration, decreasing its bioavailability, intracellular concentration, and its transition of the blood–brain barrier (BBB). P-gp, highly expressed on the endothelial cell surface contributes to reduced chemotherapeutic drug penetration to the specific sites, especially in case of brain tumor treatment where anticancer agents are generally incapable of passing through the BBB. The size of the tumor also plays a crucial role in drug penetration. Because of the poor blood supply in large tumors, chemotherapeutic agents are usually less efficient in large tumors due to the poor blood supply compared to smaller ones with nearly unlimited access of oxygen and nutrient supply. The P-gp protects the brain from potentially damaging compounds but, at the same time, restricts access of therapeutic agents responsible for higher complexity of the therapy. In most cases, the only way to overcome the barrier is to increase the concentration of the drug, which often leads to systemic toxicity. This is the reason why elevated efflux of the drug has been considered to be one of the key mechanisms of cancer cell resistance against chemotherapeutics [9,14,15,17].

P-gp and BCRP can eliminate from cancer cells a wide variety of structurally and functionally unrelated anticancer agents, including epipodophyllotoxins, anthracyclines, vinca alkaloids, bisantrene, colchicine, taxanes, imatinib, saquinavir, camptothecins, thiopurines, actinomycin D, methotrexate, and mitoxantrone to the extracellular space, reducing intracellular drug accumulation [14,15,18,19,20]. Among a variety of chemotherapeutics, significant correlation between increased expression of P-gp in cancer cells and their enhanced resistance to paclitaxel, etoposide, olaparib, DOX, and vinblastine has been found [15,21,22,23,24]. Overexpression of P-gp has been observed in about 50% of all human cancers. While, in some tumor types such as lung, liver, kidney, rectum, and colon, increased P-gp expression has been observed before chemotherapy treatment, in others, including hematological malignancies such as acute lymphoblastic leukemia and acute myeloid leukemia, overexpression of P-gp has been noticed after anticancer agents exposure [15,20]. Overexpression of P-gp and BCRP has been associated with poor clinical response and MDR in patients with multiple myeloma, acute lymphocytic leukaemia, chronic lymphocytic leukaemia, acute myelogenous leukaemia, and metastatic breast cancer [18]. Additionally, it has been reported that P-gp plays a role in cancer cells MDR not only by participating in the efflux of intracellular chemotherapeutic agents but also by inhibiting tumor necrosis factor-related apoptosis-inducing ligand TRAIL-mediated and caspase-related pathways of apoptosis [19,25,26].

Although P-gp inhibitors show a high efficacy in vitro and in vivo studies, none of them have been approved by the U.S. Food and Drug Administration (FDA) for clinical use in cancer treatment [18,27]. However, Nanayakkara et al. [27] presented some new P-gp inhibitors, which potentially may be promising drugs in cancer chemotherapy. Despite the fact that they have still not entered clinical trials, researchers using computational approach found several compounds that were able to inhibit the P-gp activity and confirmed their anticancer properties against MDR cancer cell lines. Furthermore, Nanayakkara et al. [27] tested coadministration of chemotherapeutics with the investigated compounds against two-dimensional MDR prostate and ovarian cancer cells and three-dimensional prostate cancer microtumor spheroids. The results showed a significant decrease in cell motility and cell survival and viability. Additionally, the researchers demonstrated that all of the tested P-gp inhibitors did not exhibit toxic potential and were not P-gp transport substrates. Moreover, tested compounds increased not only cellular retention of anticancer agents but also the amount of reporter compounds being P-gp transport substrates.

Another example of new potential P-gp inhibitors are naturally occurring potassium ionophores such as salinomycin. Guberović et al. [28] demonstrated that some of the investigated crown ethers revealed to be significantly more efficient in sensitising MDR cells to adriamycin and paclitaxel compared to a well-known P-gp inhibitor verapamil.

Furthermore, the results obtained by Liu et al. [29] showed that combining administration of ascorbic acid with DOX could increase the sensitivity of human MDR breast cancer (MCF-7/MDR) cells to DOX in vitro and in vivo. As those researchers showed, ascorbate improved responsiveness of the cells to DOX through promoting the cellular accumulation of the drug associated with induction of reactive oxygen species-dependent ATP depletion.

Moreover, the compound that potentially could find its application in chemotherapy treatment is tometodione M (TTM), a novel natural syncarpic acid-conjugated monoterpene. In the study of Zhou et al. [30], the drug increased intracellular rhodamine 123 and DOX accumulation in human MDR leukemia cells (K562/MDR) and MCF-7/MDR cells by decreasing P-gp-related drug efflux. TTM reduced expression of both P-gp protein and mRNA via inhibition of p38 mitogen-activated protein kinase (MAPK) signaling, leading to MDR reversion in cancer cells. Additionally, TTM not only enhanced the cytotoxicity of docetaxel in K562/MDR and MCF-7/MDR cells but also triggered apoptosis and decreased colony formation in docetaxel-treated cells [30].

In addition, the results of Yuan et al. [31] demonstrated that cinobufagin, a substance isolated from the posterior auricular glands and skin of the Asiatic toad (*Bufo gargarizans*), affected modulation of P-gp activity in human P-gp-overexpressing colorectal carcinoma cells, including Caco-2/ADR, HCT116/L, and LoVo/ADR, which could potentially find it useful in combination with chemotherapeutic agents in colon cancer treatment. Data showed that cinobufagin significantly enhanced intracellular accumulation of rhodamine 123 and DOX and exhibited apoptotic properties in MDR cells. Moreover, cinobufagin remarkably influenced P-gp overexpressing in LoVo/ADR cells by increasing their sensitivity to DOX belonging to P-gp substrate drugs. Despite the fact that further investigations on the mechanisms of action of cinobufagin showed no changes in the expression of P-gp, a significant effect of cinobufagin on noncompetitive inhibition of P-gp ATPase activity was observed [31].

Furthermore, the chemical substance which could be potentially used in cancer chemotherapy is iso-pencillixanthone A (iso-PXA), which naturally occurs in the fungus *Penicillium oxalicum*. Chen et al. [32] found that iso-PXA could increase the intracellular concentration of (VCR) in the human cervical cancer cell line HeLa/VCR by P-gp ATPase stimulation and reduction in P-gp expression. As those researchers showed, iso-PXA effectively induced the intrinsic pathway of apoptosis by poly (ADP-ribose) polymerase (PARP), caspase-3, and caspase-9 activation. Moreover, iso-PXA initiated apoptotic events by degradation of induced myeloid leukemia cell differentiation protein (Mcl-1), accumulation of F-box and WD repeat domain-containing 7 protein (FBW7), and increase of the Bax/Bcl-2 ratio.

Chen et al. [33] investigated the association between the activities of natural flavonoids, including (−)-catechin, (−)-gallocatechin, luteolin, taxifolin, and human P-gp activity. The researchers found that taxifolin in a concentration-dependent manner significantly decreased ABCB1 expression and inhibited the P-gp function through DOX efflux and uncompetitive inhibition of rhodamine 123.

Quinidine is a well-known, FDA-approved drug used clinically for the treatment of pseudobulbar effect, arrhythmia, and malaria. However, side effects of the drug associated with myocardium condition, including factors such as torsade de pointes and long QT syndrome (LQTS), complicate its clinical usage as a P-gp inhibitor. The results of Snyder et al. [34] showed potential application of polymer-drug conjugates such as methoxypolyethylene glycol (mPEG) glycine-quinidine conjugate in reversing MDR through P-gp inhibition. The investigated conjugate not only inhibited the function of P-gp comparable to quinidine but also significantly mitigated distribution of quinidine into the mouse myocardium, resulting in reduced off-target pharmacologic effects.

Sitravatinib, a novel promising receptor tyrosine kinase inhibitor, which presently is on clinical trials, has been shown to be correlated with reversing MDR of P-gp and BCRP-overexpressing cancer cells. The investigated compound inhibited the drug efflux function of P-gp and BCRP in a concentration-dependent manner without altering the protein expression of P-gp and BCRP in MDR cancer cells. As Wu et al. [18] suggested, despite the fact that sitravatinib at submicromolar concentrations reversed MDR mediated by P-gp and BCRP, further clinical trials are required.

Furthermore, the effect of novel P-gp inhibitors, polyethylene glycol-modified titanium dioxide nanoparticles (TiO_2_ PEG NPs), on cisplatin cytotoxicity against P-gp overexpressing HepG2 cells was examined. This study showed that increased cisplatin cytotoxicity was associated with downregulation of the expression of P-gp in HepG2 cells by TiO_2_ PEG NPs [35].

### 3.2. Genetic Factors

#### 3.2.1. Gene Mutations

Gene mutations, which are commonly observed in tumor cells are considered one of the main causes of the failure of chemotherapy treatment. As Duesberg et al. [36,37] concluded, the best explanation of MDR development in cancer cells is their aneuploidy nature. Researchers have suggested that frequently losing chromosomes or their reassortments during mitosis are responsible for losing drug-sensitive genes or for changes in biochemical pathways, which both seems to be crucial in chemotherapeutic drug resistance. In addition, normal cells, which rarely gain or lose a chromosome, usually stays sensitive to drugs, which makes the treatment even more complex.

**Figure 3:**
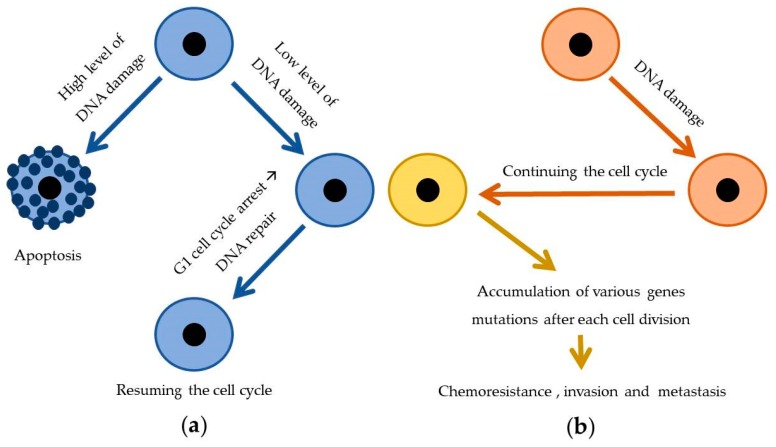
Differences in *TP53* gene expression level between normal (**a**) and cancer cells (**b**) and consequences thereof: (**a**) accurate level of expression of *TP53* gene and (**b**) decreased level of expression of *TP53* gene [38].

Mutations of the *TP53* gene, frequently observed in tumor cells, are one of the best-known biomarkers of the tumorigenesis. As Mantovani et al. [38] described, forty years of studies have demonstrated the irreplaceable role of the *TP53* gene in protecting an organism against neoplastic transformation and tumor progression. The *TP53* tumor suppressor is responsible for genome stability and cellular homeostasis by coordinating multiple processes and effector pathways, including regulation of the cell cycle and inducing apoptosis or G1 arrest in the case of any genotoxic stress caused during replication. Losing the tumor-suppressive activities by missense mutations in the *TP53* gene, which are especially widespread in human cancers, reverses the protective role of the *TP53* pathway by initiating chemoresistance, invasion, and metastasis. In a normal case, anticancer drugs, which induce DNA damage, cause cell death by *TP53* activity. In contrast, loss of the *TP53* activity in cancer cells allows continued replication no matter the type/level of DNA damage, which makes them resistant to genotoxic drugs (Figure 3) [38].

Furthermore, function of the chimeric *BCR-ABL* gene seems to be key for initiation and maintenance of tumorigenicity, especially in chronic myeloid leukemia (CML). The oncogenic gene product increases frequency of cell division, blocks DNA repair, and inhibits apoptosis. BCR-ABL tyrosine-kinase inhibitors, such as imatinib, commonly used as the first-line drug for patients with CML, prevent ATP binding to the BCR-ABL kinase receptor, therefore inducing apoptosis in cancerous cells [39,40]. Data shows that mutations of the *BCR-ABL* gene associated with the drug-binding region commonly result in imatinib resistance during the CML treatment [39]. Additionally, in some clinical studies, scientists have observed significant correlation between reactivation of the *BCR-ABL* gene and remission of CML disease [41].

Topoisomerase II-targeted agents, such as etoposide, are frequently used in order to inhibit the replication process by stopping the activity of this enzyme. Unfortunately, gene mutations of topoisomerase alter its nuclear localization, which results in the tumor cells’ resistance to the use of drug. In addition, these drugs are not specific toward cancer cells, interacting with the entire genome, which significantly limits their safe usage in cancer management [11].

The aim of cytotoxic drugs is to disable components of cells, for which the functions are key for its survival. Because of the commonly observed gene mutations in tumor cells, they are able to make some alterations in response in target molecules, which make them resistant to the specific drug. The product of the mutated gene still retains its activity, but because of some changes in its stereochemical structure, it is not able to bind to the drug anymore. A well-known example of this mechanism of resistance is antiestrogen therapy of breast cancer. Patients, who initially show proper response to tamoxifen treatment, often at some point become insensitive to an endocrine manipulation. The state of complete unresponsiveness results from the gradual loss of estrogen receptors in mutated cells. Probably, an estrogen is no longer needed for growth and functioning of survived tumor cells [42,43].

#### 3.2.2. Amplifications

The main role of many chemotherapeutics, such as methotrexate, is inhibition of key enzymes, e.g., dihydrofolate reductase engaged in controlling cell proliferation. Because of the possibility of gene amplification, which appears in 10% of the cancers, mainly in leukemias, cancer cells can overcome this inhibition by enhancing transcription of the gene, which encodes the enzyme. This process is associated with selective synthesis of a specific region of the chromosome, which provides multiple copies of the same gene. These amplified sequences are identified with homogeneously staining regions or double minute chromosomes. Each of those gene are transcribed in order to increase the mRNA level, which after that is used in the translation process to produce more enzymes. Because the drug concentration is limited, at some point, it is not able to inhibit the increased amount of enzyme [9,44].

Zhang et al. [45], using the clinically annotated genomic database, The Cancer Genome Atlas (TCGA), analyzed the transcriptomics, genomics, and clinical data of a variety of cancer samples, especially breast cancer (1082 samples). As the result, significant associations between amplification of the glycosylphosphatidylinositol-linked cell surface glycoprotein (*CD24*) gene and mutations in the *TP53* gene, cancer proliferation, and metastasis were observed. As the researchers suggested, a copy number variation of *CD24* could serve as a simple potential prognostic marker for identifying populations of interest for cancer treatment and risk subtype.

Other data present that factors such as gene rearrangements/amplification or anticancer drugs (e.g., rifampicin) could significantly increase the expression of *ABCB1* gene, leading to elevation of P-gp activity. Data from one rifampicin therapy showed that the drug increased the intestinal P-gp level 3.5-fold and decreased the oral bioavailability of another used drug (digoxin) by 30% during the whole treatment [46,47].

Genovese et al. [48] observed that even application of a single dose of chemotherapeutics, such as DOX and paclitaxel targeting cancer cells lines or hematological malignancies and various solid tumors in vivo, led to amplification of chromosome region 7q21 containing gene *ABCB1*, subsequently resulting in overexpression of P-gp and other resistance-related proteins. As the researchers pointed out, additional factors such as epigenetic modifications and single nucleotide polymorphisms (SNPs) increased expression of *ABCB1* as well. The results of a variety of studies have demonstrated that not only paclitaxel and DOX but also other anticancer agents, including anthracyclines and taxenes, caused overexpression and/or amplification of genes surrounding the *ABCB1* locus.

The upregulation of oncogene human epidermal growth factor receptor-2 (*HER2*) expression occurs in approximately 20% of breast cancers. Amplification of *HER2* leads to transcriptional modifications associated with a variety of genes and pathways in breast cancer cells. HER2 abnormal breast cancers are correlated with increased chemotherapy resistance and general worse prognosis. Anti-HER2 agents, such as lapatinib, trastuzumab, margetuximab, pertuzumab, and trastuzumab, have been used in HER2 abnormal breast cancers patients for many years. Unfortunately, administration of the inhibitor of HER2 signaling to HER2+ breast cancer patients often results in loss of initial drug sensitivity and development of resistance to used agent. The promising novel strategy of HER2 abnormal breast cancers’ clinical treatment assumes alternate combinatory agents, including cyclin-dependent kinase (CDK) 4/6 inhibitors, endocrine therapy, cholesterol pathway inhibitors, or receptor tyrosine kinase (RTK) inhibitors [49].

#### 3.2.3. Epigenetic Alterations

**Figure 4:**
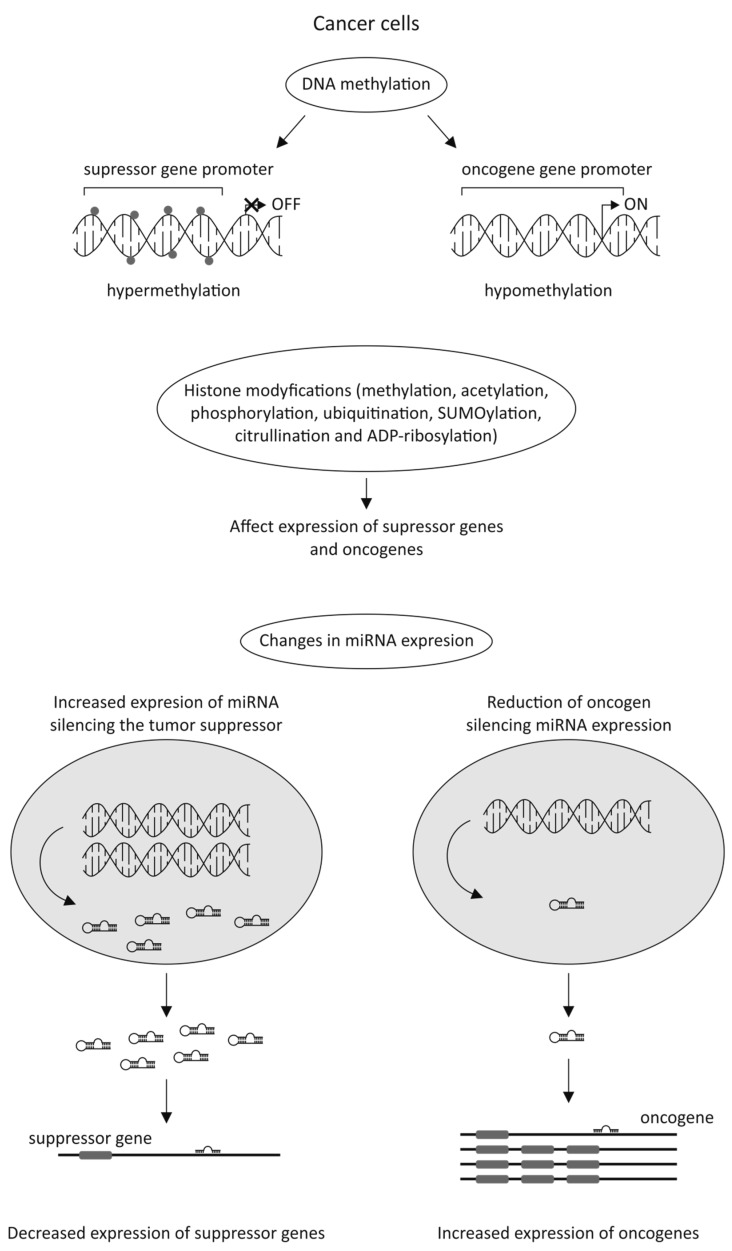
Cancer cell gene regulation by epigenetic alterations [50,51,52].

The latest data strongly emphasizes the significant role of epigenetic alterations in cancer cells in anticancer drug resistance. Silencing tumor suppressor genes by their DNA hypermethylation or enhancing the expression of oncogenes by their DNA hypomethylation could be the potential factors involved in cancer development. During tumorigenesis, the epigenome goes through multiple alterations, including genome-wide loss of DNA methylation, regional hypermethylation (especially in CpG promoter islands of tumor suppressor genes), global changes in histone modification marks, and alterations in the miRNAs expression (Figure 4) [50,51,52].

Currently, only two classes of epigenetic drugs have been approved by the FDA, i.e., DNA methylation inhibitors (iDNMT), including 5-azacitidine [53] and 5-aza-2′-deoxycytidine (decitabine; DAC) [54], as well as histone deacetylase inhibitors (iHDACs), such as Vorinostat, Belinostat, Romidepsin, and Panobinostat [55].

Demethylation of the *ABCB1* gene in the cancer cell lines leads to decreased accumulation of the anticancer agent inside the cancer cells and results in acquisition of the MDR phenotype. Furthermore, the epigenetic alterations can affect the DNA repair system. For example, hypermethylation or mutation of the human MutL homolog 1 (*hMLH1*) gene, for which the product is involved in the mismatch repair system, can result in colorectal cancer development. Data has shown that the combination of conventional chemotherapeutics and epigenetic drugs such as DAC can be an effective solution in the treatment of cancerous cells and resisted tumors. Despite the fact that DAC does not affect directly the tumor growth, it inhibits DNA methylation which sensitizes the tumor to other chemotherapeutics, including carboplatin, cisplatin, and 5-FU [44].

Development of colorectal cancer is commonly associated with a variety of epigenetic alterations, such as histone modifications, DNA methylation, noncoding RNAs, and chromatin remodeling. While DNA methylation in genes *MDF1*, *SSTR2*, *CMTM3*, *TGFB2*, and *NDRG4* is a potential marker for the detection of colorectal cancer in the early stages of its development, hypermetylation in gene *CLDN11* is associated with metastasis and poor prospect of patient survival with colorectal cancer. Furthermore, silencing of tumor suppressor candidate 3 (TUSC3) mRNA expression by promoter methylation induces signaling of epidermal growth factor receptor (EGDR), which leads to colorectal cancer cells protection from apoptosis [56]. As Patnaik and Anupriya [56] suggested, development of DNA methyltransferase inhibitors and drugs targeting histone deacetylases could be a potential novel anticancer strategy. The latest data has demonstrated that CUDC-101 and CUDC-907, new synthesized histone deacetylase/kinase inhibitors, showed therapeutic potential as anticancer agents [57,58].

Despite the fact that microRNAs (miRNAs) have only 19–25 nucleotides and are unable to code any proteins, they affect regulation of gene expression by posttranscriptional modifications. Epigenetic changes associated with miRNAs frequently play an important role in the development of chemoresistance of various types of cancer. In recent years, many studies have shown that miRNAs affect the sensitivity of tumor cells against anticancer agents by influencing drug-resistance-related genes or genes related to cell proliferation, cell cycle, and apoptosis [59]. As Mansoori et al. [44] suggested, miRNAs could serve as a biomarker for prognosis of the effectiveness of chemotherapy treatment. The list of selected miRNAs involved in tumor transformation is presented in Table 1.

### 3.3. Growth Factors

Experimental and clinical data have shown significant associations between inflammation and cancer development and progression. The results of accumulated experimental and clinical data have revealed that acute inflammation promotes tumor eradication while chronic immune response leads to tumor growth and invasion. Increased autocrine production of the growth factors, including interleukin (IL)-1, IL-4, IL-6, and IL-8, has been observed in MDR cancer cells, compared to drug-sensitive tumor cells [79,80,81,82].

It has been widely reported that IL-6 can affect various biological processes such as metabolism, differentiation, cell growth, and death by increasing *ABCB1* gene expression and the CCAAT enhancer-binding protein family activation [81]. Furthermore, Ham et al. [82] provided convincing evidence for correlation between the activity of IL-6 in cancer-associated fibroblasts occurring in the tumor stroma and MDR of gastric cancer cells. The results of the researchers showed that IL-6 was a chromatin assembly factor-1 (CAF)-specific secretory protein, which conferred gastric cancer cell chemoresistance by paracrine signaling. Moreover, they observed that application of tocilizumab, an anti-IL-6 receptor monoclonal antibody, reversed the CAF-mediated inhibition of apoptosis in both in vitro and in vivo experimental models. This data demonstrated the potential therapeutic use of IL-6 inhibitors in order to increase the responsiveness to anticancer agents in gastric cancer cells.

The results of Wang et al. [80] indicated strong association between overexpression of IL-8 in tumor tissue, serum, ovarian cyst fluid, and ascites from ovarian cancer patients and poor sensitivity for a variety of anticancer agents used during their chemotherapy. As the researchers observed, MDR in ovarian cancer cells caused by increased expression of IL-8 was associated not only with activation of Ras/MEK/ERK and PI3K/Akt signaling and overexpression of MDR-related genes, including *ABCB1* and apoptosis inhibitory proteins (XIAP, Bcl-xL, and Bcl-2) but also with decreased proteolytic activation of caspase-3. This is the reason why modulation of the IL-8 signaling pathway or IL-8 expression may be a potential strategy of MDR ovarian cancer treatment.

Cancer chemoresistance can be elevated not only by intracellular factors but also by increased level of extracellular fibroblast growth factors present in the media of metastatic and solid tumors. Data has shown that drugs with different mechanisms of action, including 5-FU, DOX, and paclitaxel, were ineffective against tumors with elevated levels of these extracellular factors. In order to prove the importance of fibroblast growth factors in development of cancer chemoresistance, Song et al. [83] applied suramin (a well-known inhibitor of these factors), which effectively reversed the 10-fold increased resistance observed in combination of intracellular and extracellular factors.

Glioblastoma, the most lethal brain cancer among adults, is a tumor characterized by marked genetic heterogeneity. However, changes in activation of receptor tyrosine kinase signaling are among the most common molecular modifications in glioblastoma. Data from a variety of studies has suggested significant association between signaling through fibroblast growth factor (FGF) receptors and glioblastoma progression. For that reason, blocking this signaling pathway by currently trialed small-molecule inhibitors of FGF receptors may be a potential strategy in glioblastoma treatment [84].

The aim of the study of Suzuki et al. [85] was to determine how extracellular FGFs affect the biology of small cell lung cancer (SCLC) cells and non-small cell lung cancer (NSCLC) cells. The results of the researchers showed significant associations between the activity of examined FGFs, i.e., FG2, FGF9, and FGF10, and proliferation, apoptosis, and treatment sensitivity of SCLC and NSCLC cells in vitro in a cell-specific manner.

Many data has shown that increased activity of protein kinase C [86] and extracellular matrix (ECM) [87] in breast tumor cells are associated with their chemotherapy resistance. It has been proven that ECM plays the key role in breast cancer progression, invasion, and metastasis. As Jena and Janjanam [87] suggested, remodeling of ECM is the major factor responsible for promoting cancer invasion and metastasis, especially matrix metalloproteinases (MMPs), including MMP-2, -9, -11, and -14, which degrade the matrix proteins. It was reported that β-D mannuronic acid could be a potential anticancer agent by inhibition of MMP-2 and -9. However, other factors such as ECM integrin b1-, b5-, and b6-; Hic-5 and ECM1 proteins; and enzymes, including heparanase, procollagen lysyl hydroxylase-2, LOXL2, and LOXL4 have also been shown to play a role in the regulation of breast cancer development and progression. Furthermore, stromal cells, including adipocytes, cancer-associated fibroblasts, and tumor-associated macrophages (TAMs) have been shown to be associated with tumor progression via a variety of processes (e.g., creating a vessel network which supports the nourishment of the tumor mass and secretion of vascular endothelial growth factor A (VEGF-A) by TAMs leading to tumor invasion) [87].

### 3.4. Increased DNA Repair Capacity

Another possibility of becoming tumor cells resistant to a variety of anticancer drugs is their ability to repair DNA damage. DNA repair endonuclease XPF and DNA excision repair protein ERCC1 involved in the nucleotide excision repair (NER) pathway are essential for the efficient repair of DNA damage induced by crosslinking and platinum-based agents [88]. A significant correlation between overexpression of both the XPF and ERCC-1 proteins and the development of cisplatin resistance in cancer cells was shown [89]. Low target specificity of a variety of anticancer drugs developed so far is the reason of their failure in chemotherapy treatment. However, successful use of PARP inhibitors against breast cancer (*BRCA*)-deficient tumors showed a new perspective on developing novel potential inhibitors of DNA repair proteins [90].

Novel compounds, i.e., E-X PPI2 and E-X AS7 have been identified as ERCC1-XPF inhibitors. Enhanced melanoma cell sensitivity to cisplatin, inhibition of NER activity, and decreased level of ERCC1-XPF heterodimers in ovarian cancer cells were observed after E-X PPI2 or E-X AS7 usage [91]. Additionally, one of the investigated catechol-based inhibitors of ERCC1-XPF (13 compound) displayed high activity in NER and selectivity against deoxyribonuclease I and Flap structure-specific endonuclease 1 (FEN-1), which resulted in enhanced cisplatin activity in A375 melanoma cells [92]. Gentile et al. [88], using a multistep computational approach, found potential modification sites of F06, an inhibitor of the ERCC1-XPF. Among investigated analogs of F06, increased IC_50_ value (0.49 µM) for the inhibition of ERCC1-XPF activity was observed in a case of B5 compound. These results require further testing and optimization; however, methods based on the computational approach described by the researchers can be used to develop novel potential ERCC1-XPF inhibitors.

Repair and tolerance of Pt-DNA lesions depend not only on NER but also on efficiency of homologous recombination (HR) pathway. Data has demonstrated that replication protein A (RPA) can be a new promising target in chemotherapy treatment. RPA, as a single-strand DNA (ssDNA)-binding protein, not only is involved in DNA recombination and replication but also plays key functions in DNA-damage response (DDR), HR, and NER DNA repair pathways [93,94,95]. In some studies, the activity of novel derivatives of RPA inhibitors against in vitro and in vivo models of NSCLC and epithelial ovarian cancer (EOC) has been examined. One of the investigated compounds, the TDRL-551, showed anticancer activity both as a single agent and in combination with Pt in an NSCLC in vivo model. In addition, synergy of TDRL-551 with platinum in both xenograft and tissue culture models of EOC was observed [93]. Furthermore, anticancer properties of one of the previously identified RPA inhibitors, SMI MCI13E, was investigated using both ovarian and lung cancer cell lines. In this study, decreasing RPA DNA-binding activity and disruption’s RPA role in the cell cycle regulation was noted. Addition of SMI MCI13E to cisplatin enhanced its anticancer properties. The results have shown that RPA small molecule inhibitors can be applied as a single chemotherapeutic or may be used in combination with current anticancer agents to enhance their efficacy [94]. Moreover, new analogues of a previously reported RPA inhibitor, TDRL-551, were designed in order to enhance physicochemical properties and anticancer activity. Compounds 43, 44, 45, and 46 were identified as chemical substances with increased solubility, low micromolar RPA inhibitory activity, and enhanced cellular uptake, holding promise for further investigation of novel chemotherapeutics [95].

Opposite to other DNA repair pathways, decreased activity of the DNA mismatch repair (MMR) pathway is associated with enhanced damage tolerance that leads to increased mutagenicity and chemoresistance. Hypermethylation of the *hMLH1* gene promoter, causing a decreased expression of the MLH1 protein involved in the MMR pathway has been found in many cancers. In vitro studies have demonstrated that 5-fluoro-2-deoxycytidine and the cytidine analog, decitabine, can reverse this hypermethylation and increase cell sensitivity to cisplatin via restoring MMR functionality [96,97].

Ataxia telangiectasia and Rad3-related protein ATR kinase plays an essential role in the regulation of the DDR pathway. Its inhibition has been shown to be associated with enhanced sensitivity of some cancer cells in vitro to DNA-damaging agents, including platinum-based compounds. However, data about successful application of ATR inhibitors in vivo is limited. Hall et al. [98] examined VX-970 anticancer properties in both in vitro and in vivo lung cancer models. The researchers observed enhanced sensitivity of most of the investigated lung cancer cell lines in vitro to a variety of DNA-damaging drugs after VX-970 usage. In vivo, in primary lung xenografts derived from patients, VX-970 inhibited ATR activity in tumors and significantly increased the efficacy of cisplatin. The combination of cisplatin and VX-970 resulted in tumor regression in a model sensitive to cisplatin and complete inhibition of tumor growth in three cisplatin-resistance models [98]. The substance AZD6738 is another ATR kinase inhibitor which induces senescence and cell death in NSCLC cells. AZD6738 increases cytotoxicity of gemcitabine and cisplatin in NSCLC cell lines and enhances cisplatin anticancer properties in ATM-deficient NSCLC cells. ATR kinase inhibition caused by daily administration of AZD6738 for 14 days was well tolerated in mice and increased the therapeutic properties of cisplatin in xenograft models. The synergy of AZD6738 and cisplatin showed strong anticancer properties against ATM-deficient lung cancer xenografts [99].

Due to interrupted HR repair in *BRCA1*-deficient breast cancer cells, DNA double-strand breaks (DSBs) in these cells can be repaired only by the nonhomologous end joining (NHEJ) pathway. This is the reason why inhibition of DNA-dependent protein kinases (DNA-PKcs) involved in DDR and NHEJ pathways could be a new promising target in *BRCA1*-deficient breast cancer treatment [100,101]. Albarakati et al. [100] observed a synergy between cisplatin and two highly selective DNA-PKcs inhibitors, NU7026 and NU7441, in *BRCA1*-deficient breast cancer cell lines.

Sustained regressions in patient-derived xenograft models after treatment with AZD7648, the highly selective DNA-PK inhibitor and efficient sensitizer of DOX, and radiotherapy (radiation-induced DNA damage) was observed. In addition, combination of AZD7648 with olaparib, a well-known PARP inhibitor, resulted in cell growth inhibition, apoptosis, and enhanced genomic instability in ATM-deficient cells model. Furthermore, AZD7648 elevated olaparib efficacy in both patient-derived xenograft and xenograft models contributing to sustained tumor regression [101].

RAD51 is a protein involved in HR pathway responsible for DNA DSB repair. It binds to ssDNA and assists in HR repair by exchanging DNA strand breaks. Enhanced HR and overexpression of RAD51 have been found in multiple myeloma cells. Furthermore, high RAD51 expression in vivo has been shown to be correlated with chemoresistance and poor patient survival. The compound B02 interrupted binding RAD51 to ssDNA, inhibiting HR pathway, which resulted in enhanced cancer sensitivity to a variety of DNA damaging agents, such as DOX. In contrast, the combination of DOX and B02 had no impact on normal human CD19+ B cells from peripheral blood [102].

In a case of DNA-damaging chemotherapy treatment, the process of mutagenic translesion synthesis (TLS) was significantly associated with development of MDR in cancer cells. Wojtaszek et al. [103] demonstrated the highly specify small-molecule inhibitor JH-RE-06 that interrupted recruitment of mutagenic POL ζ involved in TLS activity. Coadministration of JH-RE with cisplatin increased cisplatin-induced cytotoxicity both in cultured mouse and human cell lines. Previous research also revealed association between disturbing POL ζ and enhanced efficiency of DNA-lesion chemotherapy [104].

The TLS pathway is responsible for repair of inter-strand DNA cross-links (ICLs). This process is regulated by Lys-164-mono-ubiquitinated proliferating cell nuclear antigen (PCNA). Inoue et al. [105] observed that T2 amino alcohol (T2AA) inhibited TLS repair and increased DNA DSBs by interrupting the function of Lys-164-mono-ubiquitinated PCNA. Blocking the interaction between genes involved in the DNA repair, *REV1* and mono-ubiquitinated PCNA, resulted in inhibition of ICL repair and enhanced cisplatin cytotoxicity.

Mutation of TLS DNA polymerase Rev1 in cancer cells modified their TLS activity, increasing proliferating cells survival by enhancing tolerance to DNA damage occurring during replication. Sail et al. (2017) showed that two new synthesized compounds—4 and 5—inhibited mutagenic Rev1/Polζ-dependent TLS in cells, sensitizing human fibrosarcoma HT1080 cells to cisplatin. Additional experiments confirmed specificity of the investigated compounds, making them first inhibitors of TLS that target C-terminal domain of Rev1 (Rev1-CT) [106].

It has been reported that DNA DSBs in a DICER- and DROSHA-dependent manner generate DNA damage response RNAs (DDRNAs), responsible not only for the DDR management but also for guiding DNA repair. As Gioia et al. [107] observed, enoxacin, a compound being a DICER activity booster, increased the DDR signaling and DNA repair in cells exposed to ionizing radiations. Stimulation of DDRNAs production by enoxacin at dysfunctional telomeres and at chromosomal DSBs promoted accumulation of TP53 at damaged sites and, in consequence, caused suppression of homologous recombination, leading to DNA repair towards more accurate and faster nonhomologous end-joining. Unfortunately, augmented DNA repair elevated by the enoxacin not only increased the survival of normal cells but also affected cancer cells treated with anticancer agents, which might potentially result in acquisition of MDR phenotype by these cells.

### 3.5. Elevated Metabolism of Xenobiotics

As known, carrier molecules and enzymes responsible for drug metabolism play role in cancer cells chemotherapy resistance. Several studies have suggested that exposure to anticancer drugs may lead to induction and expression of gene products that protect the cell. Drug metabolizing enzymes are an integral part of phase I and phase II metabolism that helps in the detoxification of endogenous and exogenous substrates (xenobiotics).

Isoforms of cytochrome (CYP) such CYP1A6, CYP1A2, CYP1B1, CYP2C9, CYP2B6, CYP2C19, CYP3A4/5, and CYP2D6 are essential for phase I of drug metabolism and detoxification. Overexpression of CYP1B1 has been observed in various cancer cell types that modify the biotransformation of chemotherapeutics, such as mitoxantrone, flutamide, docetaxel, and paclitaxel [108]. Furthermore, increased expression of CYP2A6 enzyme, which is involved in metabolism of anticancer agents, including ifosfamide, cyclophosphamide, aflatoxin, and fluorouracil, has been reported in breast tumor tissues. In addition, highly upregulated expression of CP4Z1, CYP1B1, and CYP2A7 in cancer cells was associated with their enhanced resistance to a variety of chemotherapeutics [109].

Altered expression of enzymes involved in phase II of drug metabolism, including glutathione-*S*-transferases (GSTs), gamma-glutamyl transferases (γGTs), uridine diphospho-glucuronosyltransferases (UGTs), thiopurine methyltransferases (TPMTs), and dihydropyrimidine dehydrogenases (DPDs) in cancer cells may enhance their MDR [108,110]. The ability to inhibit the UGT activity, mainly UGT1A1, by kinase inhibitors, including sorafenib, regorafenib, pazopanib, and lapatinib, has been observed. However, in contrast to pazopanib and lapatinib activities, inhibition of UGT1A1 by sorafenib and regorafenib has been shown to be correlated with hyperbilirubinemia in patients [111]. Furthermore, novel UGT1A4 inhibitors that selectively increased sensitivity of cancer cells to chemotherapeutic agents demonstrated a new potential strategy in overcoming cancer MDR [112].

In order to overcome MDR in cancer cells with elevated GST and γGT expression, γGT-activated arsenic-based prodrugs, including darinaparsin and 4-(*N*-(*S*-glutathionylacetyl)amino)phenylarsonous acid (GSAO), as well as GST-activated agents such as nitrogen mustard have been employed [110,113]. Additionally, natural flavonoids derivatives, such as phloretin, phloridzin, baicalein, and baicalin (with micromolar concentrations), were shown to be associated with inhibition of GST activity [114]. Other novel inhibitors of GST enzymes and chalcone derivatives, including 4-methoxychalcone, 4,4′-diflurochalcone, 2′-hydroxy-4-methoxychalcone, 4′-hydroxychalcone, and 4-fluorochalcone, were also reported [115].

As FeiFei et al. [116] found, there was a significant correlation between losing an F-box only protein 8 (FBX8), a key component of the SKP1-CUL1-F-box (SCF) E3 ubiquitin ligases, and acceleration of colon tumorigenesis. FBXB, through the ubiquitination process, led to degradation of GSTP1, resulting in suppression of colorectal cancer progression.

Glutathione (GSH) functions are associated with maintaining cellular redox homeostasis. GSH detoxifies xenobiotics as well as enhances MDR in cancer cells. In contrast to normal cells, cancer cells show a higher reactive oxygen species (ROS) production. Due to vicious proliferation and enhanced metabolism in cancer cells, these cells developed an enhanced antioxidant defense system to manage the elevated oxidant state. As many data suggested, alterations in GSH level have been correlated with multiple pathways of programmed cell death in cancer cells [117,118,119]. Tumor tissues derived from lung, liver, colon, and breast cancers show overexpression of GSH compared to normal tissues. The enhanced detoxifying ability of GSH in cancer cells has been shown to be associated with decreased activity of chemotherapeutic agents [117,119,120].

The impairment of the GSH antioxidant defense system could sensitize cancer cells to currently used chemotherapeutics. It was suggested that the moderate decline in the GSH level would be an effective strategy to increase the sensitivity of cancer cells to chemotherapies [121]. The ways to deplete the cellular GSH level include the following: reduction of GSH precursor availability [122,123], inhibition of the GSH synthesis process [124], increase of GSSG levels [125], direct conjugation with GSH [110], and promotion of cellular GSH efflux [126].

## 4. Discussion

In this paper, molecular mechanisms of MDR in cancer cells have been widely described. Moreover, based on data of recent studies and discovery in the field of molecular biology, the most prospective antineoplastic agents have been presented. Highly specific molecular targets of each individual anticancer drug seemingly indicate that mutual features of these substances cannot be found. However, surprisingly, the mechanisms of action of all described (potential) chemotherapeutics are based on their inhibitory properties. Furthermore, those substances may be divided into some groups depending on their interactions with particular enzymes or other proteins involved in individual mechanisms of MDR.

The main group of these antineoplastic agents are those which interact with molecular components necessary for proper functioning of DNA repair mechanisms pathways. This includes ERCC1-XPF inhibitors (E-X PPI2, E-X AS7, compound 13, and compound B5), RPA inhibitors (TDRL-551, SMI MCI13E, and TDRL-55 derivatives), ATR kinase inhibitors (VX-970 and AZD6738), DNA-PKcs inhibitors (NU7026, NU7441, and AZD7648), HR inhibitors (B02 compound), and TLS inhibitors (JH-RE-06, T2AA, and compounds 4 and 5) [88,91,92,93,94,95,98,99,100,101,102,103,104,105,106]. Other substances, such as taxifolin, sitravatinib, cinobufagin, crown ethers, ascorbic acid, TTM, so-PXA, mPEG glycine-quinidine conjugate, and TiO_2_ PEG NPs that have been designed to block efflux of drugs outside the cancer cells are known as P-gp inhibitors [18,28,29,30,31,32,33,34,35]. Another group involves drugs that are capable of increasing metabolism of xenobiotics in tumor cells. This includes inhibitors of GST (flavonoids and chalcone derivatives) directly involved in phase II of drug metabolism [114,115]. Furthermore, growth factor inhibitors such as IL-6 inhibitors (tocilizumab) and MMP-2/-9 inhibitors (β-d mannuronic acid) have been designed to affect tumor progression, invasion, and metastasis by inhibition of chronic immune response and prevention of remodeling ECM [82,87]. The last group of novel anticancer agents like CUDC-101 and CUDC-907 are histone deacetylase/kinase inhibitors that alter expression of specific genes, for which products are involved in different mechanisms of chemotherapy resistance in cancer cells [57,58].

Currently, only Sitravatinib and CUDC-101 are on the stage of clinical trials [18,57]. The majority of previously described novel potential chemotherapeutic agents are recently discovered or synthesized. For that reason, these compounds achieved only in vitro and in vivo successful results so far, and further investigations are required. However, new trends for searching for antineoplastic agents are well visible. In spite of researchers still focused on general anticancer properties of designed drugs such as their cytotoxic or genotoxic activity, more and more studies are being conducted in order to recognize specific molecular activity of these substances that would allow to develop the strategies for overcoming MDR in tumor cells. Particularly, understanding complex mechanisms responsible for MDR in cancer cells may be a key factor in designing novel strategies of cancer treatment in future. This may include a combination of multiple specific inhibitors that together will be able to change expression of key genes associated with cancer development, to inhibit efflux of drugs outside the cell, to prolong the presence of the active form of drugs inside the cell, and to increase tumor cell sensibility to DNA damage. Due to those reasons, we can speculate that future tumor treatment strategies will be based on combination therapies that will include the use of different types of drugs that target specific weak points of particular mechanisms of MDR.

## 5. Conclusions

The development of MDR is a complex process associated with enhanced efflux of drugs, elevated metabolism of xenobiotics, increased DNA repair capacity, growth and genetic factors, or any combination of these mechanisms. Knowledge of weak points of these mechanisms enabled scientists to develop new strategies against MDR cancer cells. Among novel potential anticancer agents presented in this paper, a remarkable part of these compounds demonstrated a strong anticancer activity in single application in both in vitro and in vivo studies. However, data has shown that their combination with other drugs significantly increased efficiency of cancer treatment. This confirms the current paradigm that combination therapy is considerably more efficient compared to any one drug on its own. Due to complicated nature of the mechanisms of MDR and heterogeneity of tumor diseases, probably, there will never be an individual drug which will find its use in every type of cancer treatment. This is the reason why further efforts to investigate the mechanisms of cancer drug resistance, especially identifying their currently unknown vulnerabilities, seems to be crucial in designing novel potential chemotherapeutics. Identifying new drugs that will be able to reverse MDR in cancer cells will increase the efficiency of commonly used chemotherapeutic agents, especially on the last stages of cancer development, and will give us an opportunity to treat currently incurable tumors.

## Figures and Tables

**Table 1:** Exemplary miRNAs that play an important role in cancer resistance.

Cancer Type	miRNA	Chemotherapy Agent	Reference
prostate cancer	microRNA-34a	paclitaxel	[60]
	microRNA-217, microRNA-181b-5p	docetaxel, cabazitaxel	[61]
pancreatic cancer	microRNA-320a micro-146	5-FU	[62]
	microRNA-205, microRNA-7	gemcitabine	[63]
colorectal cancer	microRNA-519c	5-FU	[64]
	microRNAs-384	oxaliplatin	[65]
	microRNA-96	5-FU	[66]
cervical cancer	microRNA-499a	paclitaxel	[67]
	microRNA -125a	paclitaxel	[68]
	microRNA-224	paclitaxel	[69]
breast cancer	microRNA-27b-3p	tamoxifen	[70]
	microRNA-21	trastuzumab	[71]
	microRNA-134	DOX	[72]
ovarian cancer	miR-23b	paclitaxel	[73]
	microRNA-125b	paclitaxel	[74]
	microRNAs-449	DOX	[75]
gastric cancer	microRNA-508-5p	VCR, adriamycin, cisplatin, 5-FU	[76]
	microRNA-103/107	DOX	[77]
	microRNA-495-3p	adriamycin, cisplatin, 5-FU, VCR	[78]

## Abbreviations

ABC	ATP-binding cassette
ATR	Ataxia telangiectasia and Rad3-related protein
5-FU	5-Fluorouracil
BCRP	Human breast cancer resistance protein
BBB	Blood–brain barrier
BRCA	Breast cancer gene
CAF	Chromatin assembly factor-1
CD24	Glycosylphosphatidylinositol-linked cell surface glycoprotein gene
CDK 4/6	Cyclin-dependent kinase 4/6
CML	Chronic myeloid leukemia
CYP	Cytochrome
DDR	DNA-damage response
DDRNAs	DNA damage response RNAs
decitabine; DAC	5-Aza-2′-deoxycytidine
DNA-PKc	DNA-dependent protein kinase
DOX	Doxorubicin
DPDs	Dihydropyrimidine dehydrogenases
DSB	Double-strand break
ECM	Extracellular matrix
EOC	Epithelial ovarian cancer
FBW7	F-box and WD repeat domain-containing 7 protein
FBX8	F-box only protein 8
FDA	U.S. Food and Drug Administration
FEN-1	Flap structure-specific endonuclease 1
FGF	Fibroblast growth factor
GSAO	4-(*N*-(*S*-glutathionylacetyl)amino)phenylarsonous acid
GSH	Glutathione
GSTs	Glutathione-*S*-transferases
HER2	Human epidermal growth factor receptor-2 gene
hMLH1	Human MutL homolog 1 gene
HR	Homologous recombination
IC50	Half-maximum inhibitory concentration
ICLs	Interstrand DNA cross-links
iDNMT	DNA methylation inhibitor
iHDAC	Histone deacetylase inhibitor
iso-PXA	Iso-pencillixanthone A
LQTS	Long QT syndrome
MAPK	Mitogen-activated protein kinase
Mcl-1	Induced myeloid leukemia cell differentiation protein
MDR	Multidrug resistance
MMP	Matrix metalloproteinase
MMR	Mismatch repair
mPEG	Methoxypolyethylene glycol
NER	Nucleotide excision repair
NHEJ	Nonhomologous end joining pathway
NSCLC	Non-small cell lung cancer
PARP	Poly (ADP-ribose) polymerase protein
PCNA	Lys-164-mono-ubiquitinated proliferating cell nuclear antigen
P-gp; ABCB1	P-glycoprotein; ATP-binding cassette subfamily B member 1
ROS	Reactive oxygen species
RPA	Replication protein A
RTK	Receptor tyrosine kinase
SCF	SKP1-CUL1-F-box
SCLC	Small cell lung cancer
SNP	Single nucleotide polymorphism
ssDNA	Single-strand DNA
T2AA	T2 amino alcohol
TAM	Tumor-associated macrophage
TCGA	The Cancer Genome Atlas
TiO2 PEG NP	Polyethylene glycol-modified titanium dioxide nanoparticle
TLS	Translesion synthesis
TPMTs	Thiopurine methyltransferases
TRAIL	Tumor necrosis factor-related apoptosis-inducing ligand
TTM	Tometodione M
UGTs	Uridine diphospho-glucuronosyltransferases
VCR	Vincristine
VEGF-A	Vascular endothelial growth factor A
γGTs	Gamma-glutamyl transferases
